# New species of *Ancistrocerus* (Vespidae, Eumeninae) from the Neotropics with a checklist and key to all species south of the Rio Grande

**DOI:** 10.3897/zookeys.718.21096

**Published:** 2017-12-04

**Authors:** Patrick K. Piekarski, James M. Carpenter, Barbara J. Sharanowski

**Affiliations:** 1 Department of Biology, University of Central Florida, Orlando, FL 32816, USA; 2 Division of Invertebrate Zoology, American Museum of Natural History, New York, NY 10024, USA; 3 Department of Entomology, University of Manitoba, Winnipeg, MB R3T2N2, Canada

**Keywords:** Description, Eumeninae, morphology, taxonomy, Vespidae

## Abstract

A new species of potter wasp from South America, *Ancistrocerus sur* **sp. n.**, is described. A species key and checklist for all described *Ancistrocerus* that occur south of the Rio Grande are provided. New synonymy includes *Odynerus bolivianus* Brèthes = *Ancistrocerus pilosus* (de Saussure), while the subspecies *bustamente discopictus* Bequaert, *lineativentris kamloopsensis* Bequaert, *lineativentris sinopis* Bohart, *tuberculocephalussutterianus* (de Saussure), and *pilosus ecuadorianus* Bertoni, are all sunk under their respective nominotypical taxa.

## Introduction

*Ancistrocerus* is a genus of potter wasps with a solitary lifestyle, and belongs to the subfamily Eumeninae (Vespidae). Unlike eusocial vespid wasps, mothers nest alone and rear daughters without the aid of other females, and do not provision offspring progressively throughout their larval development. Since all *Ancistrocerus* presumably mass provision their progeny, their sting is specialized to paralyze and preserve prey items (Cowan 1991). They prey upon Lepidopteran, Coleopteran and Hymenopteran larvae (Iwata 1971; Yamane 1990). *Ancistrocerus* are typically tube renters, utilizing pre-existing cavities such as borings in twigs, stems and wood, abandoned mud-dauber cells, and old burrows of ground-nesting bees and wasps to build their nest (Cowan 1991; Iwata 1971; Krombein 1979; Yamane 1990), but some species make aerial mud nests (e. g. *spilogaster* Cameron, 1905, *lutonidus* Bohart, 1974, *waldenii* Viereck, 1906; see Krombein 1979). *Ancistrocerus* occurs worldwide (except Australia), and currently 116 species (You et al. 2013), with seven occurring in the Neotropics and 22 in North America (Bequaert 1925; Carpenter and Garcete-Barrett 2003; Carpenter and Genaro 2011; Krombein 1979), have been described. Currently, 12 species have been described that can be found south of the Rio Grande.

Eumeninae phylogeny and taxonomy is not well resolved and ~65% of vespid species belong to the subfamily (Carpenter and Cumming 1985; Hermes et al. 2014; Pickett and Carpenter 2010; Vernier 1997). Recently, Eumeninae was limited to include only the tribes Eumenini and Odynerini, and is comprised of a total of 3407 described species (Bank et al. 2017; Piekarski 2017). *Ancistrocerus* belongs to the tribe Odynerini in the sense of Hermes et al. (2014), but that tribe was not supported by the large-scale phylogenomic analysis by Bank et al. (2017) and Piekarski (2017). Due to their close relationship to eusocial wasps, understanding the biology and relationships among solitary potter wasps has implications for the conception of how sociality emerged (Hunt 2007). Here we present a key and checklist for *Ancistrocerus* that occur south of the Rio Grande, and describe a new species.

## Materials and methods

The specimens used in this study are deposited at the American Museum of Natural History (New York, USA). Specimens were examined under a stereomicroscope equipped with an ocular micrometer. Body length was measured from the frons to the apex of the abdomen. Photographs were taken using a Canon 7D Mark II with a Canon MP-E 65mm 1–5× Macro Photo lens. We utilized the Canon MT–24EX Macro Twin Lite for lighting, and used a custom-made diffuser to minimize hot spots. Each image is a montage of 50 layered photos that were taken using a StackShot. Photo layers were montaged using Zerene Stacker 1.04 (Zerene Systems LLC.).

Terminology follows Carpenter and Cumming (1985), and Carpenter and Garcete-Barrett (2002). Terga are referred to as T I, T II, etc.

## Taxonomy

### Ancistrocerus sur

Taxon classificationAnimaliaHymenopteraVespidae

Piekarski & Carpenter sp. n.

http://zoobank.org/878A9495-0796-4CD6-9516-1EEDFDE34BFB

#### Material examined.

Holotype. Female, ARGENT: Jujuy Posta Lozano 15–17 Dec 1967 C.C. Porter. Allotype. Male, BOLIVIA: Tarija, V-7 1969 C. Porter. Paratypes. 9 females, 12 males.

#### Diagnosis.

**Figure 1.**
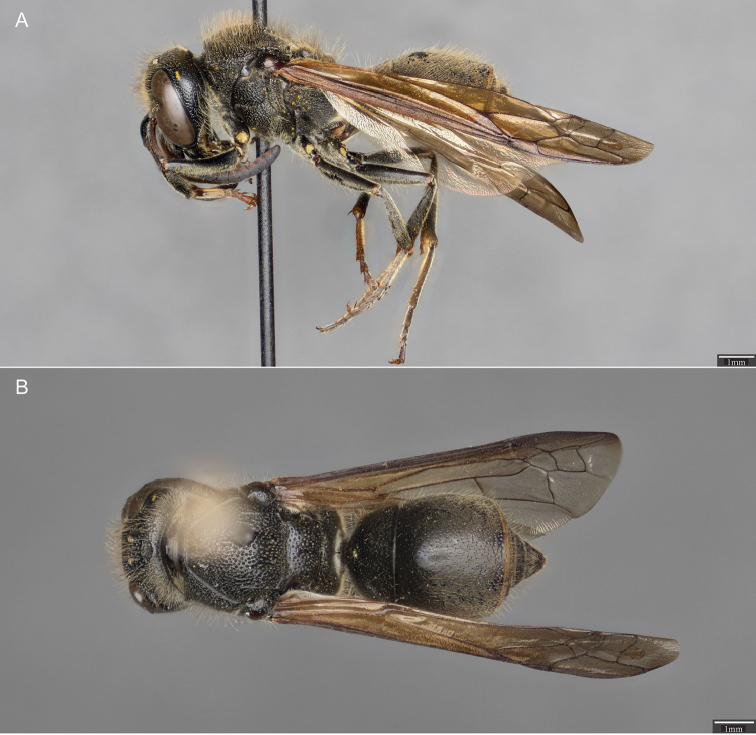
*Ancistrocerus sur*, sp. n. **A** Lateral view of the holotype (female) **B** Dorsal view of the holotype.

**Figure 2.**
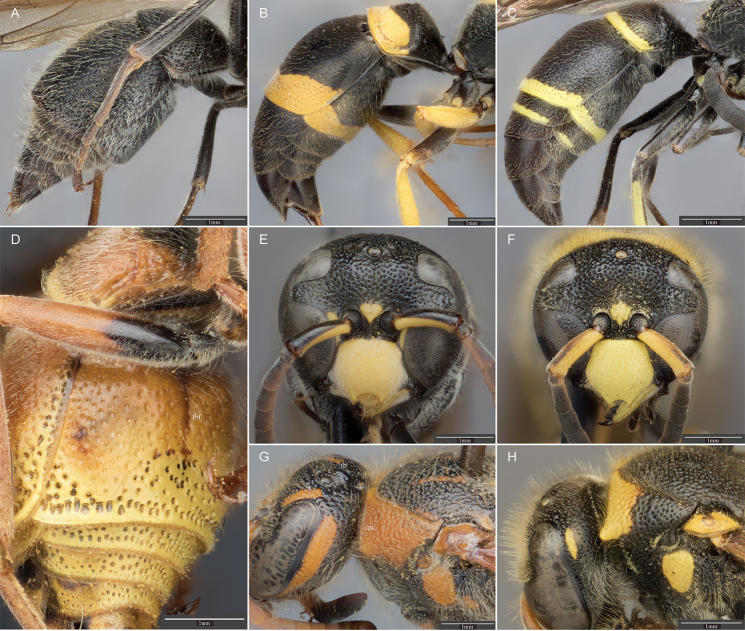
Lateral view of the metasoma of an **A** *Ancistrocerus sur* sp. n. male, with sternum II strongly truncate posterior to transverse furrow **B** *A. cingulatus* (Cresson) female, with sternum II convex posterior to transverse furrow **C** *A. santaanna* (de Saussure) male, with sternum II flat posterior to transverse furrow **D** Ventrolateral view of *A. tuberculocephalus* (de Saussure) female; sternum II with deep, longitudinal basomedian furrow. Frontal view of the clypeus for **E** *A. arista* (de Saussure) male with clypeus having a deep, semicircular emargination; and **F** *A. lineativentris* Cameron male with clypeus not having a deep, semicircular emargination. Dorsolateral view of vertex and pronotum of **G** *A. tuberculocephalus* (de Saussure) female, with pronotal carina present dorsolaterally and a polished tubercle posterior to ocelli; and **H** *A. lineativentris* Cameron male, with pronotal carina absent and vertex without tubercle. lbf = longitudinal basomedian furrow; tb = tubercle; dpc = dorsal pronotal carina.

**Figure 3.**
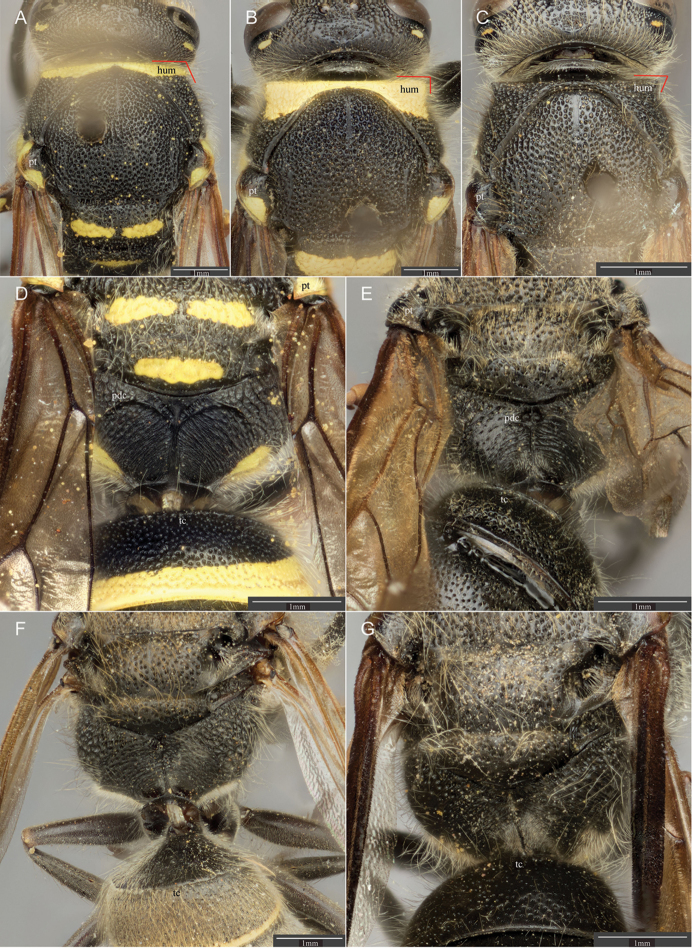
Dorsal view of mesosoma for **A** *Ancistrocerus bustamente* (de Saussure) female, with parategulae of mesonotum narrowed, and humeral angle obtuse and not projecting **B** *A. epicus* (Zavattari) female, with parategulae of mesonotum broadly flattened, and humeral angle approximately a right angle and projecting bluntly **C** *A. sur* sp. n. female, with parategulae broad and humeral angle acute and projecting sharply. Dorsoposterior view of propodeum and T I for **D** *A. bustamente* (de Saussure) female, with propodeal dorsal carina complete **E** *A. similis* (Smith) male, with propodeal dorsal carinae incomplete **F** *A. flavomarginatus* (Brèthes) female, without propodeal dorsal carina and T I with carina well developed dorsally; and **G** *A. sur* sp. n. female, without propodeal dorsal carina and tergum I with carina effaced dorsally. pt = parategula; hum = pronotal humeri; pdc = propodeal dorsal carina; tc= T I carina.

**Figure 4.**
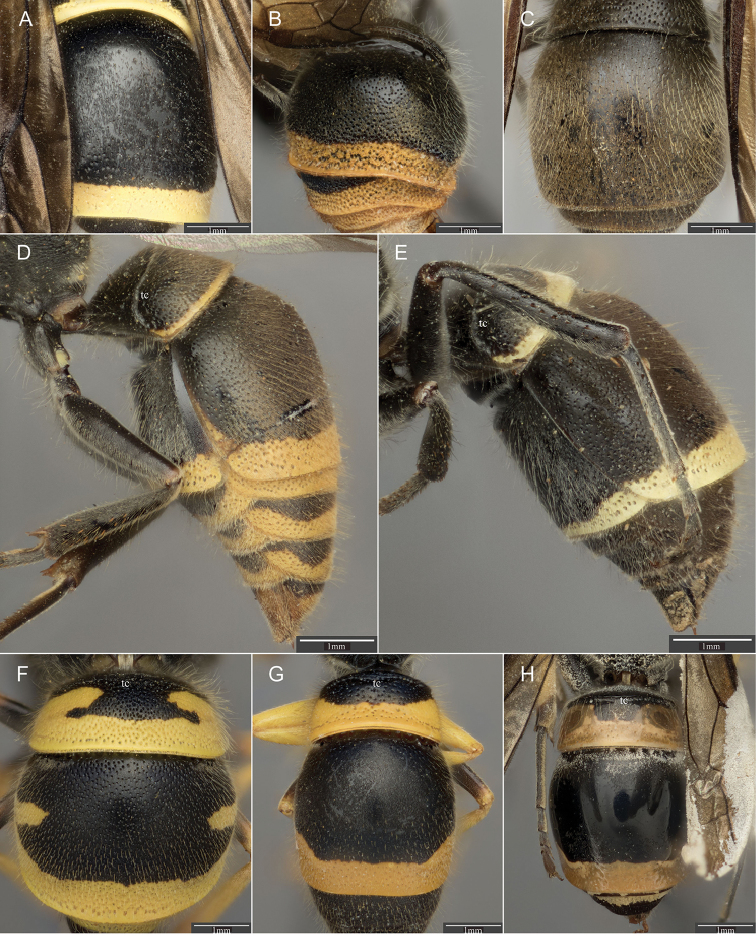
Dorsal view of T II for **A** *Ancistrocerus epicus* (Zavattari) female, with punctures small and reduced apically, and ivory maculations **B** *A. similis* (Smith) male, with punctation dense apically; and **C** *A. sur* sp. n. female, with punctation less dense apically and ending about one puncture diameter from apex. Lateral view of metasoma for **D** *A. flavomarginatus* (Brèthes) female, with metasomal maculations abundant and orange-yellow; and **E** *A. pilosus* (de Saussure) female, with pale maculations and sparse after T II. Dorsal view of T I-III for **F** *A. durangoensis* Cameron female, with punctation coarse on T II and apices slightly thickened or reflexed, and pubescence consisting of long hairs **G** *A. cingulatus* (Cresson) female, with T I and II dull, with fine punctation, and T I carina sharp and thin; and (H) *A. isla* Carpenter female, with T I and II shiny with punctures superficial, and T I carina thick and blunt. tc = T I carina.

This species can be distinguished from all other Neotropical *Ancistrocerus* using a combination of the following characters: sternum II lacking a longitudinal basomedian furrow; sternum II in lateral view strongly truncate posterior to transverse furrow (Fig. 2a); parategula broadly flattened (Fig. 3c); humeri with angular projection (Fig. 3c); T I with carina effaced dorsally (Fig. 3g); T II with punctation ending about one puncture diameter from apex (Fig. 4c); maculations reduced, on metasoma usually at most T II with a very narrow apical yellow band (Fig. 1a, b).

#### Description.

***Female.*** Body length 11.50–14.00 mm. **Color.** Almost entirely black; small traces of yellow may be present at apex of clypeus; small yellow dot in antennocular space, interantennal space, and upper gena; usually have thin, ferruginous band at apex of T II-VI and sterna II-VI (Fig. 1b). Tarsi ferruginous (Fig. 1a).

**Head**. Twelve antennal articles; 1st flagellomere ~1/3 the size of scape; pedicel ~1/2 size of 1st flagellomere; vertex with pubescence as long as distance between posterior ocelli; vertex with dense coarse punctures, much less dense than on clypeus; vertex without tubercle; clypeus about as long as wide, narrowed apically with slight concavity at tip; mandibles decussate, four teeth spaced along the edge; mandibular ridges present; antennal sockets less than 1/2 socket diameter away from clypeus; palpal formula 6:4; maxillary palpomere two about same length as palpomere three; a narrow interantennal distance, approximately the diameter of a antennal socket; ocello-occipital distance greater than the length of the ocellar triangle; cephalic foveae closely spaced, set in a slight medial depression which is delimited posteriorly by a carina; dorsal occipital carina simple and complete, without fork, running to mandible; gena most wide dorsally.

**Mesosoma**. Long thoracic hairs (Fig. 1a); puncture density similar throughout (except tegula and anterior pronotal face); anterior pronotal face largely impunctate, and without paired medial foveae; lateral pronotal foveae present; pronotal carina weaker on dorsum; humeral carina absent, but sharp angular projection at the humeri (Fig. 3c); pretegular carina present; epicnemial carina absent; no apparent notaulices and parapsidal furrows; tegula without large punctures, appearing smooth; tegula tapered posteriorly, reaching slightly beyond the parategula; parategula broadly flattened (Fig. 3c); axillary fossa oval, broader than long; metonotum rounded and sloped; metanotum without tubercles; propodeum without complete dorsal carinae (Fig. 3g); propodeum without shelf and sloping posteroventrally; propodeal valvula rounded, and not free posteriorly. **Wings.** Prestigma less than half length of pterostigma; marginal cell distally rounded with small appendix; both recurrent veins received by second submarginal cell; basal angle of second submarginal cell acute; second submarginal cell not petiolate. **Legs.** One midtibial spur; bifid tarsal claws.

**Metasoma.** Thin white or yellowish hairs on metasoma, longest on T I; T I carina effaced dorsally (Fig. 3g); width of T1 at least twice as long as wide; T1 without apical lamella; T II with very thin apical lamella; T II with punctation ending about one puncture diameter from apex (Fig. 4c); T I and T II punctation equally dense, but T II punctures slightly smaller; apices of terga not more punctate than rest of terga; bottom of basal sulcus with longitudinal ridges; sternum II in lateral view strongly truncate posterior to transverse furrow (Fig. 2a); sternum II without basomedian longitudinal sulcus; sterna with similar puncture size and density as corresponding terga.

**Figure 5.**
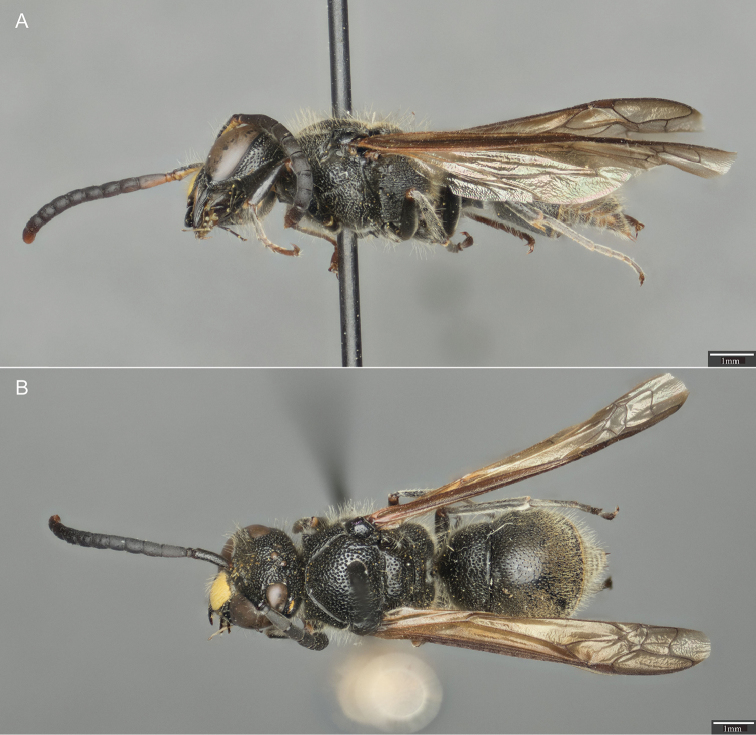
*Ancistrocerus sur*, sp. n. **A** Lateral view of the allotype (male) **B** Dorsal view of the allotype.

***Male.*** Body length 10.00–13.00 mm. **Color.** Almost entirely black; clypeus usually entirely yellow (Fig. 5b); scape may be yellow ventrally; mandible may have yellow traces; small yellow dot present on upper gena but typically absent in antennocular and interantennal space; usually have ferruginous band at apex of T II-VII and sterna II-VII (Fig. 5b). Tarsi ferruginous.

**Head.** Identical to female, except for: 13 antennal articles; apex of antennae hooked; clypeus longer than wide, narrowed apically with slight concavity at tip; mandibles decussate, four (five on allotype) teeth spaced along the edge; cephalic foveae absent.

**Mesosoma.** Identical to female (Fig. 5a, b).

**Metasoma.** Identical to female, but 7 metasomal segments and male genitalia. T II apex in male not reflexed (cf. *A. arista* and *A. similis*).

#### Distribution.

Argentina, Bolivia.

#### Etymology.

The name is the Spanish word for “south,” referring to its southerly distribution in the Neotropics. It is to be treated as a noun in apposition.

##### Key to the species of *Ancistrocerus* south of the Rio Grande

**Table:** 

1	Sternum II with deep, longitudinal basomedian furrow at least one third the length of the sternum (Fig. 2d); T II usually coarsely punctate apically	**2**
–	Sternum II lacking longitudinal basomedian furrow, or if a shallow one is present it is less than one third the length of the sternum; T II coarsely punctate apically or not	**4**
2	Pubescence on scutum fine, less than one ocellus diameter long; color gray with orange-yellow maculations; clypeus with deep, semicircular emargination (Fig. 2e)	***arista* (de Saussure)**
–	Pubescence on scutum longer than one ocellus diameter (Fig. 1a); color not gray; clypeus without deep, semicircular emargination (Fig. 2f)	**3**
3	Pronotal carina present dorsolaterally (Fig. 2g); vertex with large, polished tubercle posterior to ocelli (Fig. 2g) which may be reduced in some females	***berculocephalus* (de Saussure)**
–	Pronotal carina absent; vertex without tubercle (Fig. 2h)	***lineativentris* Cameron**
4	Sternum II in lateral view flat or slightly concave posterior to transverse furrow (Fig. 2c)	***santaanna* (de Saussure)**
–	Sternum II in lateral view evenly convex or strongly truncate posterior to transverse furrow	**5**
5	Sternum II in lateral view strongly truncate posterior to transverse furrow (Fig. 2a)	**6**
–	Sternum II in lateral view evenly convex posterior to transverse furrow (Fig. 2b)	**11**
6	Parategula narrow (Fig. 3a); propodeum with dorsal carinae (Fig. 3d); humeral angle obtuse and humeri not projecting (Fig. 3a); upper part of temple uniformly punctate; male clypeus emarginate apically, about as wide as long	***bustamente* (de Saussure)**
–	Parategula broadly flattened (Fig. 3b, 3c); propodeum without dorsal carinae (Fig. 3e), or partially present but sublaterally incomplete; humeri with blunt (Fig. 3b) or sharp projections (Fig. 3c); upper part of gena with few large punctures beneath spot; male clypeus truncate apically, length greater than width	**7**
7	T II with punctation reduced apically, punctures small (Fig. 4a); humeri with blunt projections (Fig. 3b); maculations ivory-yellow	***epicus* (Zavattari)**
–	T II with punctures as large or larger apically than rest of tergum, even if punctation is reduced; humeri with sharply angular projections (Fig. 3c); maculations pale to orange yellow	**8**
8	T II with punctation dense apically (Fig. 4b), male with apex reflexed	***similis* (Smith)**
–	T II with punctation less dense apically (Fig. 4c), often absent adjoining apex	9
9	T I with carina effaced dorsally (Fig. 3g); T II with punctation ending about one puncture diameter from apex (Fig. 4c); maculations reduced, on metasoma usually at most T II with a very narrow apical yellow band (Fig. 1b)	***sur* Piekarski & Carpenter, sp. n.**
–	T I with carina well developed dorsally (Fig. 3f); T II with punctation either extending to apex, or ending several puncture diameters before it; maculations rarely so reduced	**10**
10	Maculations abundant, orange-yellow; all terga and sterna after II with bands (Fig. 4d) but no line on pronotum	***flavomarginatus* (Brèthes)**
–	Maculations variable in extent, pale yellow (Fig. 4e)	***pilosus* (de Saussure)**
11	Punctation coarse on mesosoma; T II and III with punctation dense, coarser near apices than on rest of surface, apices slightly thickened or reflexed (Fig. 4f); pubescence consisting of long hairs (Fig. 4f) (Rocky Mountains, New Mexico, Texas)	***durangoensis* Cameron**
–	Punctation fine on humeri and scutum, nearly absent on metasoma; pubescence reduced (Greater Antilles)	**12**
12	T I and II dull, with fine punctation (Fig. 4g); T I carina sharp, thin (Fig. 4g) (Cuba)	***cingulatus* (Cresson)**
–	T I and II shiny (Fig. 4h), punctures superficial, appearing almost impunctate; T I with carina blunt, thick (Fig. 4h) (Puerto Rico)	***isla* Carpenter**

## Discussion

Color variability is usually a poor character to demarcate species due to large variability within and between closely related species, and because distantly related species occupying the same area share similar coloration patterns (Richards 1978). However, all described neotropical species, except *Ancistrocerus sur*, have distinct colored maculations on the mesosoma and/or metasoma. A darker gestalt is always in combination with the proposed diagnostic characters of *Ancistrocerus sur*, including sternum II strongly truncate posterior to transverse furrow, an effaced dorsal carina on tergum I and a projecting acute humeral angle. Although, the extent of coloration in the female clypeus varied, the lack of colored maculations on the mesosoma and metasoma is consistent across Bolivian and Argentine representatives. Thus, a lack of coloration on the metasoma and mesosoma is a reliable diagnostic character for *Ancistrocerus sur*.

There exists sexual dimorphism in clypeus color between males and females of *Ancistrocerus sur*, as well as presence/absence of yellow spots in the antennocular and interantennal space. Males lack a T2 with a reflexed apex, suggesting that the sexual dimorphism in this species may be less than in other *Ancistrocerus*. Typically, male vespids either have the same number of teeth as conspecific females, or fewer (Carpenter 1988b; c; Carpenter and Cumming 1985). Unusually, based on our examined material, it seems that females of *A. sur* have four mandibular teeth, while males tend to have five (some appearing to have four). Within *Ancistrocerus* there is variability as to what characters are sexually dimorphic across species. Thus, this genus may be an exceptional group for studying how traits diverge across sexes.

### Checklist

Genus *Ancistrocerus* Wesmael

*Ancistrocerus* Wesmael, 1836, Bull. Acad. R. Belg. 3: 45, subgenus of *Odynerus* Latreille.

Type species: *Vespa parietum*, Linnaeus, 1758, by subsequent designation of Girard, 1879, Traité Élem. d’Entomol. II (2): 900.

*Euancistrocerus* Dalla Torre, 1904, Gen. Ins. 19: 36, name for division II of subgenus Ancistrocerus Wesmael of genus *Odynerus* Latreille in de Saussure, Ét. Fam. Vesp. 1: 127, 3: 209.

Type species: *Vespa parietum* Linnaeus, 1758, subsequent designation of van der Vecht and Carpenter, 1990, Zool. Verh. Leiden 260: 21.

Valid species: 116 spp.

Distr.: Ethiopian (22 spp.), Nearctic (22 spp.), Neotropical (7 spp.), Oriental (18 spp.) and Palearctic Region (57 spp.)

**Diagnosis.** Male antenna hooked apically; female cephalic foveae closely spaced, sometimes in slight depression, nearer occipital margin than posterior ocelli, but not in distinct area of differentiated cuticle; anterior face of pronotum without medial pits or foveae (cf. *Parancistrocerus*); pronotal carina weak or absent dorsally, but well developed laterally; pronotum without oblique humeral carina (cf. *Parancistrocerus*); pretegular carina present; tegula tapered posteriorly, reaching slightly beyond the parategula; axillary fossa oval, broader than long; epicnemial carina absent; midtibia with one spur; metanotum somewhat flat, without tubercles; dorsal face of propodeum short or lacking, and sloped; propodeal concavity divided by well-developed median longitudinal carina; propodeal valvula not enlarged; metasoma sessile, not petiolate; T1 with width more than half that of T2 in dorsal view, and T1 less than twice as long as wide; T1 with single transverse carina near summit; T1 without broad longitudinal median groove posterior to carina (c.f. *Symmorphus*); sternum II with basal transverse furrow; prestigma length < one-third stigma; second recurrent vein received by second submarginal cell; second submarginal cell not petiolate.

*arista* (de Saussure)

*Odynerus arista* de Saussure, 1857, Rev. Mag. Zool. (2) 9: 274, sex not stated (in subgenus Ancistrocerus, division *Ancistrocerus*) – “Mexique: Cuernavaca” (Genève).

Distr.: Mexico: Morelos.

*bustamente* (de Saussure)

*Odynerus Bustamente* de Saussure, 1857, Rev. Mag. Zool. (2) 9: 273, sex not stated (in subgenus Ancistrocerus, division *Ancistrocerus*) – “De Pérote, au Mexique” (lectotype female Genève).

*Odynerus Bustamenti* de Saussure, 1875, Smithson. Misc. Coll. 254: 157 (key), 172. Unjustified emendation.

*Odynerus pictiventris* Cameron, 1906, Trans. Am. Entomol. Soc. 39: 331, female – “New Mexico” (type depository unknown).

*Ancistrocerus neocallosus* Bequaert, 1944, Entomol. Amer. (N. S.) 23: 236, 239 (key), 264, fig. 3, female, male – “Arizona: Mt. Lemmon, Sa. Catalina Mts., Pima Co., 6,000 ft.” (holotype female Cambridge); also from numerous other localities; and TX, KS, CO, UT, NV, CA.

*Ancistrocerus neocallosus* var. (or subsp.) *discopictus* Bequaert, 1944, Entomol. Amer. (N. S.) 23: 236, 268, female, male – “California: Round Valley, San Jacinto Mts., Riverside Co., 9,200 ft.” (holotype female Cambridge); also from numerous other localities; and AZ. REVISED STATUS.

Distr.: U.S.A.: TX, KS, CO, UT, NV, CA, AZ, NM; Mexico: Veracruz.

Note: The *discopictus* is a minor color variant, like other cases in Eumeninae discussed by Carpenter (1988a, 2003) and Carpenter and van der Vecht (1991). In its description Bequaert (1944: 269) mentioned that both it and the nominotypical form occurred in the same locality, and were connected by transitional specimens. Bequaert stated that “It may, nevertheless, be useful to distinguish the variety by name, as it parallels similar color forms of other species of *Ancistrocerus* in the same area” but we disagree; formal nomina are a poor way to deal with continuous variation. We therefore synonymize it.

*cingulatus* (Cresson)

*Odynerus cingulatus* Cresson, 1865, Proc. Entomol. Soc. Philad. 4: 162, female – “Cuba” (coll. Gundlach, Habana).

Distr.: Cuba.

*durangoensis* Cameron

*Ancistrocerus durangoensis* Cameron, 1908, Trans. Am. Entomol. Soc. 34: 216, male – “Durango, Colorado” (Zürich).

*Ancistrocerus fulvicarpus* Cameron, 1908, Trans. Am. Entomol. Soc. 34: 222, female – “South-west Colorado” (Zürich).

“*Ancistrocerus behrensi* Cr.” Tucker, 1909, Trans. Kans. Acad. Sci. 22: 286 – “Colorado, Buffalo.” *Nomen nudum*.

Distr.: U. S. A.: OK, TX, NM, AZ, UT, CO, WY; Mexico: Chihuahua.

*epicus* (Zavattari)

*Odynerus epicus* Zavattari, 1912, Arch. Naturgesch. 78A (4): 174 (key), 191, female (in subgenus Ancistrocerus, division *Euancistrocerus*) – “Peru: San Paulo” (coll. Magretti, Milano; *recte*: Torino).

Distr.: Peru.

*flavomarginatus* (Brèthes)

*Odynerus flavomarginatus* Brèthes, 1906, An. Mus. Nac. Buenos Aires 13: 349, 371 (key), female (in subgenus Ancistrocerus) – “Brasil” (Buenos Aires, Montevideo).

Distr.: Brazil; Paraguay.

*isla* Carpenter

*Ancistrocerus isla* Carpenter, 2011, in Carpenter & Genaro, Insect. Mund. 0202: 1, 5 (key), 6, figs. 25-26, 41, female – “Puerto Rico: Mayaguez” (Washington); also from another locality.

Distr.: Puerto Rico.

*lineativentris* Cameron

*Ancistrocerus lineativentris* Cameron, 1906, Invert. Pacif. 1: 146, male – “Mountains near Claremont, California” (Pomona, no. 3949).

*Ancistrocerus lineativentris* var. (or subsp.) *kamloopsensis* Bequaert, 1944, Entomol. Amer. (N. S.) 23: 280, male, female – “British Columbia: Kamloops” (holotype female Cambridge); also from U. S. A.: OR, WY. REVISED STATUS.

Ancistrocerus lineativentris var. fulvicarpus; Bequaert, 1944, Entomol. Amer. (N. S.) 23: 236, 279 (key), 281. Misidentification.

*Ancistrocerus lineativentris sinopis* Bohart, 1974, in Bohart & Menke, J. Kans. Entomol. Soc. 47: 466, male, female – “Mt. Lemmon Lodge, Santa Catalina Mts., Arizona” (holotype male Davis); also from numerous other localities; and CO, TX, UT. REVISED STATUS.

Distr.: Canada: B. C.; Western U. S. A. east to SD, KS; Mexico: Chihuahua.

Note: The *kamloopsensis* and *lineativentris sinopis* are both minor color variants, which are known to intergrade with the nominotypical form (Bequaert, 1944: 280), and we therefore synonymize them.

*pilosus* (de Saussure)

*Odynerus pilosus* de Saussure, 1855, Ét. Fam. Vesp. 3: 218, male (in subgenus Ancistrocerus) – “Le Perou” (Paris).

Ancistrocerus pilosus var. ecuadorianus Bertoni, 1918, An. Cient. Parag. (2) 3: 197, female – “Santa Inés, Ecuador” (San Lorenzo). REVISED STATUS.

*Odynerus bolivianus* Brèthes, 1920 (1919), Ann. Soc. Entomol. Fr. 88: 397, female (in subgenus Euancistrocerus) – “Bolivia: Beni” (Buenos Aires). NEW SYNONYMY.

Distr.: Venezuela; Colombia; Peru; Ecuador; Bolivia.

Note: One of us (JMC) has seen the types of both *pilosus* and *bolivianus*, and a specimen of *pilosus var. ecuadorianus* from the Bertoni collection in San Lorenzo that is probably a type. Female *pilosus* compare very well to the type female of *bolivianus* and are very similar in coloration, and we herewith synonymize these taxa. The variety *ecuadorianus* is a minor color variant, and is also synonymized.

*santaanna* (de Saussure)

*Odynerus Santa-anna* de Saussure, 1857, Rev. Mag. Zool. (2) 9: 273, female, male (in subgenus Ancistrocerus, division *Ancistrocerus*) – “Le Mexique” (Genève).

*Odynerus Santa-annae* de Saussure, 1875, Smithson. Misc. Coll. 254: 159 (key), 171. Unjustified emendation.

Distr.: Mexico: Veracruz, Jalapa, Michoacán.

*similis* (Smith)

*Odynerus similis* Smith, 1857 (April), Cat. Hym. Brit. Mus. 5: 80, female – “Mexico” (London).

*Odynerus Parredes* de Saussure, 1857 (June), Rev. Mag. Zool. (2) 9: 273, sex not stated (in subgenus Ancistrocerus, division *Ancistrocerus*) – “Le Mexique, Mextitlan” (Genève).

*Odynerus Parredesi* de Saussure, 1875, Smithson. Misc. Coll. 254: 158 (key), 180. Unjustified emendation.

*Odynerus pilosellus* Cameron, 1912, Timehri 2: 221, female – “Costa Rica” (London).

Distr.: U. S. A.; Mexico: Hidalgo, Nuevo León, Tamaulipas; Guatemala; Costa Rica; Panama.

*tuberculocephalus* (de Saussure)

*Odynerus Tuberculocephalus* de Saussure, 1852, Ét. Fam. Vesp. 1: 122 (key); 1853: 139, pl. XVI fig. 9, male, female (in subgenus Ancistrocerus) – “Le Mexique” (Genève?).

*Odynerus tuberculiceps* de Saussure, 1853, Ét. Fam. Vesp. 1: Errata and Explanation of pl. XVI fig. 9. Unjustified emendation.

*Odynerus sutterianus* de Saussure, 1875, Smithson. Misc. Coll. 254: 186, male, female (in subgenus Ancistrocerus) – “California” (Genève).

“*Odynerus nigrohirsutulus* Cameron” Bequaert, 1925, Trans. Am. Entomol. Soc. 51: 114 (label on possible type of *A.* (?) *nigro-hirsutus* Cameron; belongs in *Ancistrocerus*). *Nomen nudum*.

Ancistrocerus tuberculiceps var. sutterianus Bequaert, 1944, Entomol. Amer. (N. S.) 23: 236, 283 (key), 284. REVISED STATUS.

Distr.: Canada: B. C.; U. S. A.: CA, NV, UT, ID, OR, SD, WY, UT, CO, AZ, NM, TX; Mexico: Chihuahua, Hidalgo, Jalisco, Michocan, DF, Tamaulipas, Veracruz.

Note: The subspecies sutterianus is a minor color variant, which is known to intergrade and to co-occur with the nominotypical form (Bequaert 1944: 283), and we therefore synonymize it.

## Supplementary Material

XML Treatment for Ancistrocerus sur
